# Interest in Determining the CD34+ CD38− Phenotype in the Diagnosis and Prognosis of Acute Leukemia in Abidjan – Côte d’Ivoire

**DOI:** 10.4084/MJHID.2013.023

**Published:** 2013-04-10

**Authors:** Duni Sawadogo, Aissata Tolo, Hermance Kassi, Mahawa Sangare, Andre Inwoley

**Affiliations:** 1 Department of Hematology. Faculty of Pharmacy. University Felix Houphouet Boigny. Cocody. Abidjan. Unit of Hematology. Central Laboratory. Teaching Hospital of Yopougon. Abidjan; 2 Clinical hematology department- Teaching Hospital of Yopougon - Abidjan; 3 Hematology unit- Central Laboratory-Teaching Hospital of Yopougon - Abidjan; 4 Department of hematology, immunology and cellular biology - Faculty of pharmacy- University Felix Houphouet Boigny. Abidjan. Hematology unit- Central Laboratory-Teaching Hospital of Yopougon – Abidjan.; 5 Department of hematology, immunology and cellular biology - Faculty of pharmacy- University Felix Houphouet Boigny. Abidjan. Immunology laboratory, Center for the study and research on AIDS and opportunistic diseases (CeDReS) - Teaching Hospital of Treichville- Abidjan.

## Abstract

**Background:**

In Côte d’Ivoire, acute leukemias account for 12.5% of hematological malignancies. Acute leukemias are due to an anomaly of the stem cell characterized among other things by the expression of CD34^+^ CD38^−^ surface markers. This CD34^+^ CD38^−^ phenotype as well as other factors such as tumor syndrome, high leukocytosis and blasts are considered as important factors of poor prognosis. We therefore proposed to investigate the prognostic value of the expression of CD34^+^ CD38^−^ markers in acute leukemias in Abidjan.

**Methods:**

We selected 23 patients aged 33 years on whom we performed Complete Blood Count, bone marrow aspiration and immunophenotyping. To search for myeloperoxydase, smears of blood or bone marrow were stained with benzidine and revealed by the use of Hydrogen peroxide. Acute leukemias were then identified and distributed using the score proposed by the European Group for the Immunological characterization of Leukemias. The definitive diagnosis was made by combining morphological characters that serve as the basis for the French-American-British classification as well as cytochemical and immunophenotypic characters.

**Results:**

According to the cytological and immunophenotypic classifications, the acute lymphoid leukemia 2 and B IV predominated. 52.2% (12/33) of patients were CD34^+^ CD38^−^. This phenotype was found in almost all cytological immunophenotypic types. The medullary invasion by blasts (reflection of the tumor mass) of the total sample of CD34^+^, CD34^+^ CD38^−^ patients and those not expressing CD34^+^ was respectively 79.4%, 81.25%, 83.3% and 74.8%.

**Conclusion:**

There was therefore no correlation between medullary blasts and the expression of CD34^+^ CD38^−^. To the factors we selected it would have been necessary to associate the study of cytogenetic and molecular anomalies to better understand the role of CD34^+^ CD38^−^ phenotype, concerning prognosis.

## Introduction

In Côte d’Ivoire, cancers in general and hematological malignancies in particular are booming due to urbanization and HIV-AIDS.^1^–^3^ According to Tanon,^3^ non-Hodgkin lymphomas were associated with HIV infection but not with leukemia.^3^ Acute leukemia (AL) accounted for 12.5% of hematological malignancies and were more related with pollution and environment.^1^,^2^

Factors such as tumor syndrome, high leukocytosis and blasts are considered as factors of poor prognosis. However they are not the only ones. Indeed Basso^4^ and George^5^ pointed out that AL is a stem cell disease in which the stem cell self-renewal mechanisms are preserved but the tight growth control is lost due to malignant transformation. Leukemia stem cells are thought to reside within the CD34^+^ CD38^−^ population.^5^–^10^ CD34 has been frequently associated with a worse prognosis and a poor outcome in AL. Indeed the expression of P 170 glycoprotein on leukemic cells confers them resistance to chemotherapy or ≪Multiple Drug Resistance (MDR)≫. The expression of MDR is related to CD34 phenotype.^4^,^9^,^10^ Conventional chemotherapy, based treatment of leukemia and cancer in general, is primarily directed against the bulk of malignant cells and thus does not eliminate the abnormal stem cells. Those cells are the origin of cancer recurrence and are responsible for relapse.^4^–^10^

The purpose of this study was to investigate the prognostic value of CD34^+^ CD38^−^ expression on leukemic blasts cells in patients with AL in Abidjan.

## Material and methods

We selected patients presenting clinical signs (alteration of the general condition, tumor, anemic, hemorrhagic, infectious syndromes) or biological signs (cytopenia, leukocytosis, myelemia) in favor of AL. After obtaining their consent, we collected blood in a tube containing ethylene diamine-tetra-acetate and/or marrow on lithium heparinate. We performed a Complete Blood Count and blood smear stained with May Gründwald Giemsa. To search for myeloperoxydase (MPo), smears of blood or bone marrow were stained with benzidine and revealed by the use of Hydrogen peroxide (H_2_O_2_). The reaction was positive when at least 3% of blasts contained in their cytoplasm blackish blue granulations.^11^ Immunophenotyping by flow cytometry was performed on the cytometer FACSCalibur KD 394 of Becton Dickinson to 1 laser with whole blood or marrow without separation of cells on Ficoll hypaque. We used Becton Dickinson’s sorting tests associated with several fluorochromes: fluorescein isothiocyanate, phycoerythrin, chlorophyll peridine protein. The monoclonal antibodies used were as follows: for B lymphoid lineage (CD45/CD38/CD56/CD19 and CD103/CD22/CD20), for T lymphoid lineage (CD45/CD3/CD4), for erythrocytic and megakaryocytic lineages (CD45/anti-glycophorin-A/CD41), for immaturity markers (anti HLA-DR/CD34/CD38). For each sample we resorted to the technique of triple labeling. After performing a windowing on blast cells with the CD45 antigen, CD45- blasts were analyzed by kits CD38/CD56/CD19 and CD103/CD22/CD20 or CD45/CD3/CD4 to determine the proliferation lineage. Antigen was considered positive when the expression reached at least 30%.^12^,^13^

AL were then identified and distributed using the score proposed by the European Group for the Immunological characterization of Leukemia (EGIL).^12^,^13^ The definitive diagnosis was made by combining morphological characters that serve as the basis for the French-American-British classification (FAB) as well as cytochemical and immunophenotypic characters.

In acute bi-phenotypic leukemia (Tor B or B/T myeloid) blasts carried both antigens of B and T lineage and antigens of myeloid and lymphoid lineages.

Acute bi-clonal leukemia was characterized by the presence of two morphologically different blastic populations with expression of different antigens. A blastic population should be either positive to benzidine (Mpo^+^) and carry T or B lineage antigens. AL undifferentiated was characterized only by cells carriers of immaturity antigens (HLA-DR, CD34 CD38). Unspecified AL cells lacked specific lineage antigens.^11^–^13^ Immunophenotyping helped to identify patients’ CD34^+^ CD38^−^.

Patients received induction chemotherapy with a combination of cytarabine and daunorubicine in acute myeloid leukemia (AML). In acute lymphoid leukemia (ALL) the treatment included the administration of steroid, vincristine, daunorubicine, asparaginase and methotrexate. Both are considered gold standard in induction therapy.

## Results

We received 72 patients with hematological malignancies. 59.7% (43/72) of patients presented lymphoproliferative disease. AL concerned 31.9 % (23/72) of this population. The main characteristics are shown in table 1. They were HIV negative. AL occurred at any age, but there was a peak frequency between 0 and 20 years. The average age of the population with hematological malignancies on the one hand and lymphoproliferative disease on the other hand was respectively 39 (range 1 to 90) and 42.02 (range 1 to 88) years.

The alteration of the general condition (65.2%) and tumor syndrome (60.9%) were the most frequently encountered clinical signs.

95.7% of patients were anemic and 34.8% presented leukocytosis superior to 50 G/l. Medullar blasts were very important at the time of diagnosis (table 1). Medullar invasion by blasts was significant and superior or equal to 80% in 15/23 patients (65.2%). More than half of patients (52.1%) presented ALL. ALL2 was the cytological type of the FAB classification most encountered. We did not find any megakaryocytic or erythrocytic AL. 17.4% of AL could not be identified accurately based on morphological and cytochemical criteria (table 1). EGIL classification demonstrated the clear predominance of ALL B IV (tables 1 and 2).

Immunophenotyping also revealed the presence of AL with ambiguous lineage. It was on the one hand about undifferentiated AL (HLA-DR and/or CD34, CD38) whose morphology and markers do not allow assignment to a lymphoid or myeloid lineage, on the other hand it was about bi-phenotypic AL (T/B about bi-clonal AL (lymphoblastic/mono-blastic). The immunophenotypic profile of each patient is collected in table 2.

52.2% (12/23) of patients were CD34+ CD38−. This phenotype was found in almost all the cytological and immunophenotypic types (table 2). CD34+ CD38− patients had respectively a white blood cell and blasts rate of 52.4 ± 79.3 G/L and 83.3 ± 16.5%. CD34+ CD38− phenotype is found in all patients regardless of value and presented poor prognosis factors such as leukocytosis, blasts (table 2).

Only 12/23 (52.2%) patients could be treated and received conventional chemotherapy. We reported in table 3 the results of the induction treatment. A complete remission was achieved in 1/12 (8.33%) patients. No one reached 5 years of disease-free survival.

## Discussion

AL is characterized by a proliferation of immature hematopoietic cells starting in the bone marrow. Like others African authors,^1^,^14^,^15^ we found a slight male predominance (table 1). Patients were young adults with an average age of 33 ± 21.7 years (table 1). This result was similar to Koffi.^14^ He worked on the results of the induction treatment in AL, in Abidjan. His patients were 28 years old (range 3 to 54).

Patients were HIV negative. Indeed, Tanon^3^ highlighted that cancer was a growing co-morbidity among HIV-infected patients in Côte d’Ivoire and Benin in 2012. With the scale-up of antiretroviral therapy in developing countries, cancer will contribute more and more to the HIV/AIDS disease burden. According to Tanon,^3^ Kaposi sarcoma, non-Hodgkin lymphoma, cervical cancer, anogenital cancer and liver cancer were all associated with HIV infection but not with leukemia.

The late presentation of patients to the hospital and the poor access to care explained the importance of the tumor mass that resulted in significant leukocytosis and lymphoid and myeloid/B lymphoid) and finally it was medullar blasts with respective values of 56.7 ± 74.2 G/l and 79.4 ± 20.7% and thrombopenia (table 1).

Unlike Koffi^14^ and Braham Jmili,^15^ who had described in their series a predominance of ALL_1_, we found that ALL_2_ were in majority (table 1). We did not diagnose any erythrocytic leukemia or megakaryocytic leukemia. They are uncommon in the African series^1^,^11^,^14^,^15^ (table 1). With cytology and cytochemistry 17.3% of AL could not be classified. These results were comparable to the ones of Braham Jmili^15^ who found 19.1% of non classifiable AL by cytological study. Secondary AL, ALL and those with lineage ambiguity such as bi-clonal AL, bi-phenotypic or undifferentiated AL were poor prognosis. They accounted for 26% of the sample. Glycoprotein CD34^+^ was found in 69% (16/23) of patients. These results were slightly superior to those of Basso^4^ who found that 25 to 64% of patients were carriers of CD34. AL expressing CD34^+^ CD38^−^ phenotype corresponded to 52.2% (12/23) of the sample (table 2). According to many authors,^4^–^10^ leukemic stem cells exist in this blastic population CD34^+^ CD38^−^. The mean medullar blasts of the sample was 79.4%, those of CD34^+^, CD34^+^ CD38^−^ patients and those not expressing CD34^+^ were respectively 81.25%, 83.3% and 74.8% (tables 1 and 2). These values were close to each other. It seemed that there was no correlation between medullar blasts and the expression of CD34+ CD38−.

52.2% (12/23) of patients could be treated and received conventional chemotherapy (table 3). The high cost of drugs, the low income, the lack of insurance and social security for population represented a serious difficulty for the treatment of the patients. This situation was also described by Ly.^17^ 83.3% (10/12) of patients died during or after the induction treatment. Only 1/12(8.3%) patients achieved complete remission (table 3). This result, at first sight, was not in agreement with Koffi^14^ who found 60% of complete remission in AL. However, a little deeper study allows realizing that many patients were excluded in this study. Indeed, only 45 patients were enrolled over a period of 5 years while in one year we recruited 23 patients in the same hospital albeit at different times. Improved diagnostic techniques may have also led to obtain a larger sample.

Ebinger,^6^ Vergez,^8^ Witte^9^ suggested that in ALL and in AML, the proportion of CD34^+^ CD38^−^ at the diagnosis may serve as a prognosis marker as well. A higher proportion of CD34^+^ CD38^−^ correlated with unfavorable prognosis. In our study, the expression of CD34^+^CD38^−^ in blasts seemed to have no influence on the results of the induction treatment. And we could not come to the same conclusion particularly for 2 reasons. On the one hand, the majority of the patients died very early after the induction therapy. On the other hand, we did not notify the proportion of CD34^+^CD38^−^ cell of each patient. We just identified the CD34^+^ CD38^−^ blasts population.

A part from CD34^+^ CD38^−^ phenotype, there were other important factors for prognosis and treatment such as cytogenetic and molecular anomalies. Vergez^8^ in AML, found that a proportion of CD34^+^ CD38^−/low^ CD123^+^ cells was greater than 15% at the diagnosis and unfavorable karyotype were significantly correlated with the lack of complete response. Chauchan^16^ investigated the expression of MDR1 and apoptotic (p53) genes in AL. MDR 1 expression was significantly associated with the expression of immature stem cell marker CD 34.

The leukemia initiating cells was found within the CD34^+^CD38^−^ cell compartment. These more immature cells were more resistant to therapy.^6^–^10^ Resistance to chemotherapy is a major impediment to the successful treatment in AL. The expression of genes involved in drug resistance and apoptosis could be responsible for this. Multidrug transporter genes such as MDR1 lead to a rapid clearance of cytotoxic drugs.^7^–^10^,^15^

As we did not perform cytogenetic and molecular studies we could not link up the results of the induction treatment and the presence or the absence of leukemic stem cell CD34^+^CD38^−^ population with the MDR genes expression (table 3). We could just conclude that patients had a very bad prognosis and outcome regardless of the expression of CD34^+^CD38^−^. Sonoda^18^ successfully identified human hematopoietic stem cells. This opens the door to a possible existence of leukemic stem cells CD34^−/low^. This hypothesis would perhaps also explain our results.

## Conclusion

AL is considered as a hematological malignancy with anomalies of CD34^+^ CD38^−^ leukemic stem cells. This CD34^+^ CD38^−^ phenotype is also in relation with the expression of multidrug resistance genes.

CD34^+^ CD38^−^ phenotype was found in patients regardless of other epidemiological (age, sex), clinical (tumor syndrome) or biological (blood and/or medullar blasts) prognostic factors. Most of the patients died without or after the induction treatment. To the factors that we studied it would have been necessary to associate the study of cytogenetic and molecular anomalies to better understand the role of CD34^+^ CD38^−^ phenotype concerning prognosis. Though this study shows limits, it is one of the rarest studies about expression of CD34^+^ CD38^−^ in AL in sub Saharan Africa.

## Figures and Tables

**Table 1:** Patient’s characteristics

Epidemiologic and biological characteristics		
Age (years)	33 ± 21.7	(2–71)
Sex (H/F)	13/10	1.3
Hb g/l	83 ± 25	(36 – 173)
WBC G/l	56.7 ± 74.2	(14 – 252. 6)
Platelets G/l	94.9 ± 85.8	(9 – 394)
Blasts %	79. 04 ± 20.7	(32 – 99)
Blasts MPo+	8/23	(35%)
		
**French American Britannic classification (FAB)**	n	%
ALL_1_	3	13.1%
ALL_2_	8	34.8%
ALL_3_	1	4.3%
AML_2_	3	13.1%
AML_4_	1	4.3%
AML_5_	3	13.1%
AL	4	17.3%
Total	23	100%
		
**Immunological classification**	n	%
ALL B I or Pro B	2	8.7
ALL B IV or B mature	10	43.5
ALL T	1	4.3
AL Biphenotypic	2	8.7
AL Biclonal	1	4.3
AML	6	26.1
AL indetermine	1	4.3
Total	23	100

**Table 2:** Epidemiological, clinic and biological characteristics

N°	Age (y)	Sex (M/F)	Tumoral syndrome	WBC (G/l)	Blasts %	FAB	MPo	Immunophenotypic profile	EGIL
1	25	H	Absent	1.4	91	ALL_2_		HLA-DR^+^/**CD34**^+^**/**CD22^+^/CD20^+^/CD19^+^/**CD38**^−^	ALL B IV
2	15	F	Present	27. 1	90	ALL_2_		CD3^+^/CD4^+^/**CD34**^+^**/**CD22^+^/CD20^+^/CD19^+^/**CD38**^−^	Biphenotypic L (B/T)
3	66	H	Present	4	98	ALL_2_	+	CD22^+^/CD20^+^/CD19^+^/CD34^−^/CD38^−^	ALL B IV
4	37	F	Absent	24.7	83	AML_2_		HLA-DR^+^/**CD34**^+^**/**CD38^+^	AML
5	53	F	Absent	2	81	ALL _2_	+	CD22^+^/CD20^+^/CD34^−^/CD38^+^	ALL B IV
6*	39	H	Present	4 .8	77	AML_4_	+	CD4^+^/HLA-DR^+^/**CD34**^+^**/CD38**^−^	AML
7	48	H	Absent	31.7	53	AML_2_		**CD34**^+^**/**CD22^+^/**CD38**^−^	Biphenotypic L (Myeloid/B)
8	54	F	Present	47	61	AL		CD22^+^/CD20^+^/CD19^+^/CD34^−^/CD38^−^	ALL B IV
9	42	H	Absent	252. 6	42	ALL_1_		CD3^+^/CD34^−^/CD38^+^	ALL T
10	71	H	Absent	73.9	86	AL		HLA-DR^+^/CD22^+^/CD20^+^/CD34^−^/CD38^+^/CD19^+^	ALL B IV
11	18	H	Present	109.1	32	AML_5_	+	HLA-DR^+^/**CD34**^+^**/**CD38^+^	AML
12	27	F	Present	5. 2	93	ALL_2_		**CD34**^+^/CD22^+^/CD20^+^/CD19^+^/**CD38**^−^	ALL B IV
13	35	H	Present	26. 7	98	ALL_2_		HLA-DR^+^/**CD34**^+^**/**CD22^+^/CD38^+^	ALL BI
14	14	F	Present	2.1	89	ALL_2_		HLA-DR^+^/**CD34**^+^**/**CD22^+^/CD20^+^/CD19^+^/**CD38**^−^	ALL B IV
15	05	H	Present	173	95	ALL_2_		HLA-DR^+^/**CD34**^+^**/**CD22^+^/**CD38**^−^	ALL B I
16	51	F	Absent	13	71	ALL_1_		HLA-DR^+^/**CD34**^+^**/**CD38^+^	Undifferentiated AL
17	13	H	Present	1. 9	99	ALL_1_		HLA-DR^+^/**CD34**^+^**/**CD22^+^/CD20^+^/CD38^+^/CD19^+^	ALL B IV
18	09	H	Present	5.7	96	ALL_3_		HLA-DR^+^/**CD34**^+^**/**CD22^+^/CD20^+^/CD19^+^/**CD38**^−^	ALL B IV
19	02	H	Absent	15. 5	96	AML_5_	+	HLA-DR^+^/**CD34**^+^**/CD38**^−^	AML
20	55	F	Present	79.9	90	AL		HLA-DR^+^/CD22^+^/CD20^+^/CD34^−^/CD38^+^/CD19^+^	ALL B IV
21	63	F	Absent	89.8	90	AML_2_	+	HLA-DR^+^/**CD34**^+^**/CD38**^−^	AML
22	05	F	Present	248	47	AML_5_	+	**CD34**^+^/CD56^+^/**CD38**^−^	AML
23	13	H	Present	66	60	AL	+	CD22^+^/CD20^+^/CD19^+^/CD34^−^/CD38^−^	AL Biclonal (Lymphoid/monoblastic)

*: secondary AL

**Table 3:** Results of the induction treatment according to the expression of CD34^+^ CD38^−^

N°	FAB	EGIL	Immunophenotypic profile	Results of the induction treatment	Outcome after12 months of follow-up
1	ALL_2_	B-lineage ALL	**CD34**^+^/**CD38**^−^	Failure	Death after induction treatment
4	AML_2_	AML	**CD34**^+^**/**CD38^+^	Failure	Death during induction treatment
5	ALL _2_	B-lineage ALL	CD34^−^/CD38^+^	Failure	Death during induction treatment
8	AL	B-lineage ALL	CD34^−^/CD38^−^	Failure	Alive
9	ALL_1_	T-lineage ALL	CD34^−^/CD38^+^	Failure	Death during induction treatment
10	AL	B-lineage ALL	CD34^−^/CD38^+^	Failure	Alive
12	ALL_2_	B-lineage ALL	**CD34**^+^/**CD38**^−^	Failure	Death after induction treatment
13	ALL_2_	B-lineage ALL	**CD34**^+^/CD38^+^	Failure	Death during induction treatment
14	ALL_2_	B-lineage ALL	**CD34**^+^/**CD38**^−^	Complete remission	Death after relapse
17	ALL_1_	B-lineage ALL	**CD34**^+^/CD38^+^	Failure	Death after induction treatment
18	ALL_3_	B-lineage ALL	**CD34**^+^/**CD38**^−^	Failure	Death after induction treatment
23	AL	AL Biclonal	CD34^−^/CD38^−^	Failure	Death during induction treatment
